# Age and Sex Differences in Hearts of Soluble Epoxide Hydrolase Null Mice

**DOI:** 10.3389/fphys.2020.00048

**Published:** 2020-02-07

**Authors:** K. Lockhart Jamieson, Hedieh Keshavarz-Bahaghighat, Ahmed M. Darwesh, Deanna K. Sosnowski, John M. Seubert

**Affiliations:** ^1^Faculty of Pharmacy and Pharmaceutical Sciences, University of Alberta, Edmonton, AB, Canada; ^2^Department of Pharmacology, Faculty of Medicine and Dentistry, University of Alberta, Edmonton, AB, Canada

**Keywords:** aging, mitochondria, soluble epoxide hydrolase, Sirt-3, cardiac function, sexual dimorphism

## Abstract

Biological aging is an inevitable part of life that has intrigued individuals for millennia. The progressive decline in biological systems impacts cardiac function and increases vulnerability to stress contributing to morbidity and mortality in aged individuals. Yet, our understanding of the molecular, biochemical and physiological mechanisms of aging as well as sex differences is limited. There is growing evidence indicating CYP450 epoxygenase-mediated metabolites of n–3 and n–6 polyunsaturated fatty acids (PUFAs) are active lipid mediators regulating cardiac homeostasis. These epoxy metabolites are rapidly hydrolyzed and inactivated by the soluble epoxide hydrolase (sEH). The current study characterized cardiac function in young and aged sEH null mice compared to the corresponding wild-type (WT) mice. All aged mice had significantly increased cardiac hypertrophy, except in aged female sEH null mice. Cardiac function as assessed by echocardiography demonstrated a marked decline in aged WT mice, notably significant decreases in ejection fraction and fractional shortening in both sexes. Interestingly, aged female sEH null mice had preserved systolic function, while aged male sEH null mice had preserved diastolic function compared to aged WT mice. Assessment of cardiac mitochondria demonstrated an increased expression of acetyl Mn-SOD levels that correlated with decreased Sirt-3 activity in aged WT males and females. Conversely, aged sEH null mice had preserved Sirt-3 activity and better mitochondrial ultrastructure compared to WT mice. Consistent with these changes, the activity level of SOD significantly decreased in WT animals but was preserved in aged sEH null animals. Markers of oxidative stress demonstrated age-related increase in protein carbonyl levels in WT and sEH null male mice. Together, these data highlight novel cardiac phenotypes from sEH null mice demonstrating a sexual dimorphic pattern of aging in the heart.

## Introduction

The prevalence of cardiovascular disease (CVD) has markedly increased as the global population ages (North and Sinclair, 2012; Benjamin et al., 2019). Important age-associated changes resulting in structural deterioration and progressive decline in cardiac function is characterized with development of left ventricular hypertrophy, systolic, and diastolic dysfunction and decreased exercise capacity (Chiao and Rabinovitch, 2015). Although the influence of age on the heart is well-documented, the sex-specific patterns of cardiac aging in males and females are less appreciated (Merz and Cheng, 2016). Sex-associated differences, such as a higher incidence of obstructive diseases in males compared to microvascular complications in females, contribute to the variations in cardiac outcomes persistently observed between men and women (Zhou and Gao, 2010; Keller and Howlett, 2016). Much of the early work into these sex-associated cardiovascular outcomes focused on the role of endogenous hormones as mediators of cardiovascular protection (Huang and Kaley, 2004). Recent data suggest that hormonal changes alone are insufficient to fully explain these variations, and other involved biological mechanisms remain a subject of ongoing debate (Regitz-Zagrosek and Kararigas, 2016).

Mitochondrial dysfunction and increased oxidative stress have been identified as key participants in cardiac aging and associated CVD (Martín-Fernández and Gredilla, 2016). Sirtuin 3 (Sirt-3) is a deacetylase enzyme primarily localized in the mitochondria involved in regulating several physiological and pathophysiological processes, including redox homeostasis, through the deacetylation and activation of various proteins (Kong et al., 2010; Benigni et al., 2016). Sirt-3 directly activates the major mitochondrial antioxidant enzyme manganese superoxide dismutase (MnSOD), which scavenges reactive oxygen species (ROS) (Sundaresan et al., 2016). Evidence has shown Sirt-3 may be down regulated during cardiac aging resulting in suppressed MnSOD activity leading to increased ROS levels (Kincaid and Bossy-Wetzel, 2013). Subsequently, increased ROS levels can activate downstream targets, including the PI3K/Akt pathway, further exacerbating age-related cardiac hypertrophic response (Kincaid and Bossy-Wetzel, 2013; Matsushima and Sadoshima, 2015; Pillai et al., 2015). Understanding the exact role Sirt-3 has in the aging processes remains a focus of many research groups trying to uncover key pathways and therapeutic approaches to treat age-related complications (Sundaresan et al., 2009; Hafner et al., 2010; Hebert et al., 2013; Porter et al., 2014).

Polyunsaturated fatty acids (PUFAs) are metabolized through numerous metabolic pathways, including the cyclooxygenase, lipooxygenase, and cytochrome P450 (CYP) monooxygenase pathways (Jamieson et al., 2017a). These transformations produce a plethora of lipid mediators with numerous biological functions (Ai et al., 2009; Lee et al., 2010; Akhnokh et al., 2016). Oxidative metabolism of PUFAs can produce bioactive mediators, termed oxylipids, which are further metabolized to less bioactive diols by the epoxide hydrolase family of enzymes (EH) (Imig and Hammock, 2009; Nithipatikom et al., 2014). Located primarily in the cytosol, the soluble form (sEH), has been implicated in the progression of multiple CVDs, including hypertension and atherosclerosis (Harris and Hammock, 2013). The microsomal form (mEH) is also an established xenobiotic-metabolizing enzyme responsible for the biotransformation of active metabolites (Marowsky et al., 2016). While mEH is capable of hydrolyzing PUFA derivatives, it has been determined to have limited roles in cardiac metabolism (Marowsky et al., 2009; Decker et al., 2012). Cardiac sEH primarily metabolizes oxylipid mediators to less active metabolites, which often results in loss of cardioprotective properties (He et al., 2016). Both genetic deletion and pharmacological inhibition of sEH has been demonstrated to mediate cardioprotective, anti-inflammatory and anti-hypertensive responses, as well limit mitochondrial injury (Jamieson et al., 2017a, b; Darwesh et al., 2019). In humans, genetic polymorphisms increasing sEH activity are associated with poor outcomes in cardiac and renal disease, although this seems to be population-dependent (Fava et al., 2010; Zhu et al., 2015; Shuey et al., 2017). Numerous animal studies have demonstrated the importance of sEH in various models of CVD; however, there is limited information regarding its role in generalized cardiac aging (Seubert et al., 2006; Monti et al., 2008; Zhang et al., 2008; Akhnokh et al., 2016; Jamieson et al., 2017b). Moreover, there is limited information regarding sexual disparity in cardiac sEH with age (Pinot et al., 1995; Sinal et al., 2000), as such the present study investigated the impact of sEH in age- and sex-dependent cardiac differences.

## MATERIALS AND METHODS

### Animals

A colony of mice with targeted deletion of the *Ephx2* gene (sEH null) with their WT littermates are maintained at the University of Alberta. Mice are conserved on a C57BL6 background. All experiments were carried out on male and female mice aged 2–4 months old (young) and 15–18 months old (middle-aged). The middle-age range, referred to hereafter as “aged,” was chosen to be clinically representative of the manifestation of CVD in humans and to avoid confounding effects of frailty, which can drastically change cardiovascular phenotypes in elderly mice (Whitehead et al., 2014). At the appropriate age, hearts and kidneys were excised from mice following euthanasia with 100mg/kg of sodium pentobarbital. Hearts and kidneys were then rinsed in 1X PBS, flash frozen in liquid nitrogen and stored at −80°C awaiting analysis. Animal experimental protocols were approved by the University of Alberta Health Sciences Welfare Committee and were carried out in accordance with the guidelines set by the Canadian Council of Animal Care.

### Cardiac Function

Transthoracic 2D echocardiography was used for cardiac functional assessment 1 week prior to animal euthanasia. Animals were anesthetized by isoflurane (1–2%) and recordings were taken using Vevo 3100 high-resolution imaging system, 40 MHz transducer (MX550S; Visual Sonics). Visual Sonics VevoLab software was used for assessment of the cardiac images. Left ventricular interior volumes and left ventricular internal diameters (LVID) were determined from m-mode images taken at the mid-papillary level. Left ventricular ejection fraction (%EF) was calculated using the equation % EF = [(LVEDV - LVESV)/LVEDV] × 100. Left ventricular mass (corrected, mg) was calculated with 1.05 × [(LVID; d + LVPW; d + IVS; d)^3-LVID; d^3] × 0.8. The transmitral filling pattern was assessed by pulsed-wave Doppler imaging to determine diastolic function. The E/A ratio represents early transmitral wave (E-wave) followed by the late filling wave due to atrial “kick” (A-wave). Tissue Doppler imaging was used to describe the motion of the mitral annulus (E’ and A’).

### Protein Expression and Immunoblot Analysis

Western blot analysis was used to determine protein expression in subcellular mitochondrial, microsomal, and cytosolic fractions. Briefly, hearts and kidneys were homogenized in ice cold homogenization buffer (250 mM sucrose, 10 mM Tris–HCL, 1mM EDTA, 1 mM sodium orthovanadate, 1 mM sodium fluoride, 10 μg/L aproptinin, 2 μg/L leupeptin and 100 μg/L pepstatin) and centrifuged at 700 × *g* for 10 min. The supernatant was then centrifuged at 10 000 × *g* for 20 min and the subsequent pellet containing mitochondria was resuspended in 70 μL homogenization buffer. The resultant supernatant was centrifuged at 100, 000 × *g* for 60 min with the supernatant taken as the cytosolic fraction and the pellet taken and resuspended as the microsomal fraction. Protein levels were quantified in subcellular fractions using standard Bradford assay. Samples containing 35 μg protein were loaded on 4–15% TGX^®^ gels (BioRad, CAN) and used for SDS-PAGE gel electrophoresis, then transferred onto 0.2 μm PVDF membranes for subsequent western blotting. Probing was done using primary antibodies against sEH (1:500, Elabscience; E-AB-60489), total-Akt (1:1000, Cell Signaling; CS9272S), phospho-Akt (Ser473) (1:1000, Cell Signaling; CS5106S), Sirt-3 (1:1000, Cell Signaling; CS5490S), total MnSOD (1:5000, Abcam; ab13533), acetyl-MnSOD (1:5000, Abcam; ab13707), α-tubulin (1:1000, Abcam, ab4074), mEH (1:200, Santa Cruz, sc135984), GAPDH (1:1000, Cell Signaling; CS2118S), and VDAC (1:1000, Abcam; ab14734). After washing with 1X TBST, membranes were incubated with the corresponding horseradish peroxidase-conjugated secondary antibodies (1:5000) and visualized with ECL reagent. The densitometry analysis was performed based on relative band intensities using Image J software (NIH, United States).

### Enzymatic Assays

Sirt-3 activity was detected in the isolated mitochondrial fractions using a Sirt-3 fluorescent assay kit (BPS Bioscience, San Diego, CA, United States), according to the manufacturer’s instructions. In this assay, mitochondria were first isolated from the hearts of young and aged male and female WT and sEH null mice. Mitochondria fractions were mixed with the specific HDAC fluorogenic substrate, bovine serum albumin, NAD^+^ and assay buffer. The deacetylation process induced by Sirt-3 in the sample sensitizes the HDAC substrate so that subsequent treatment with the Sirt-3 assay developer produces a fluorescence product that was measured using a fluorescence plate reader at 350/460 nm excitation/emission wavelengths. The activity of Sirt-3 was expressed as U/μg protein (Bochaton et al., 2015; Zhao et al., 2019).

As an established biomarker of mitochondrial content, citrate synthase activity was measured spectrophotometrically as previously described (Akhnokh et al., 2016). Briefly, heart tissues were ground and homogenized in ice-cold homogenization buffer (20 mM Tris, 40 mM KCl, 2 mM EGTA, pH7.4, with 50 mM sucrose added the day of homogenization) and centrifuged at 600 × *g* for 10 min. The supernatant was used to assess enzymatic activity spectrophotometrically as described previously (Spinazzi et al., 2012).

SOD activity was measured in the cytosolic fractions using a spectrophotometry based assay dependent upon the competition for superoxide anion (O_2_^–^) by cytochrome c and SOD. The assay utilized xanthine and xanthine oxidase as the primary source of O_2_^–^. In this assay, one unit of SOD is equal to the amount of the enzyme which inhibits 50% of the rate of the reduction of cytochrome c (Grapo et al., 1978; Beyer and Fridovich, 1987).

Protein carbonyl content was assessed in cytosolic fractions based on a reaction with 2,4-dinitrophenylhydrazine derivatization (DNPH) using a protein carbonyl ELISA kit (Abcam; Ab1238536) following manufacturer specifications.

### Mitochondrial Ultrastructure

Conventional transmission electron microscopy (TEM) was used to assess mitochondrial ultrastructure. A 1–2mm^3^ sample of myocardial tissue was obtained mid-level from the left ventricular free wall and fixed at 4°C overnight in 3% glutaraldehyde and 3% paraformaldehyde. A mixture of 1.5% potassium ferrocyanide [K_4_Fe (CN)] and 2% osmium tetroxide (OsO_4_) in 0.1M cacodylate buffer was used as a post-fixative followed by staining en bloc with 2% uranyl acetate (pH 5.2) for 1 h. Tissues were dehydrated in a continuous series of ethyl alcohol (30, 50, 70, 80, 90, 95, and 100%) followed by acetone. Resin infiltration was obtained with serial dilutions of acetone:Spurr’s resin (2:1; 1:1; 2:1; absolute Spurrs’s resin). The samples were then thermally polymerized for 24 h at 70°C, followed by ultra-thin sectioning (70 nm thickness) using an ultramicrotome (Leica UC7, Leica Microsystems Inc., Vienna, Austria). Samples were post-stained with 4% uranyl acetate and Reinolds’ lead citrate for 30 min followed by carbon-coating (Leica EM ACE600, Leica Microsystems Inc., Vienna, Austria). Sections were imaged at 60 kV using a transmission electron microscope (Hitachi H-7650 TEM, Hitachi High-Technologies Canada, Inc) equipped with a 16 megapixel EMCCD camera (XR111, Advanced Microscopy Technique, MA, United States) within 1 week of post-staining.

### Statistical Analysis

Data were expressed as mean ± standard error of mean (SEM). Statistical significance (*P* < 0.05) was determined by three-way ANOVA with Tukey’s *post hoc* test. Statistical analysis was performed using GraphPad Prism 8 software (San Diego, CA, United States). Proportional variance (eta^2^, η^2^) obtained from three-way ANOVA is represented as a factor of 1, delineating contribution of age, sex or genotype to data set variability. Significance of variance and resultant interactions was set at *P* < 0.05.

## RESULTS

### Age-Related Cardiac Hypertrophy Is Prevented in Aged sEH Null Female Mice

**FIGURE 1:**
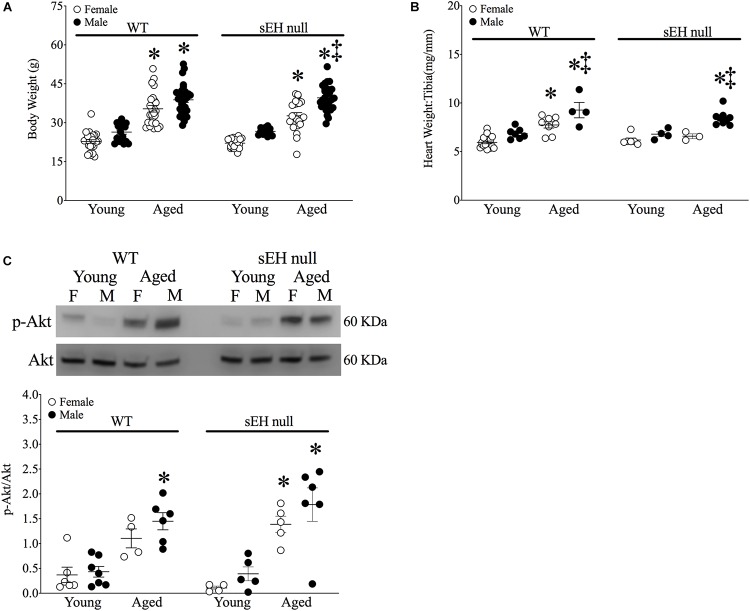
Physiological parameters in young and aged WT and sEH null mice. **(A)** Body weight (g) of mice, **(B)** Heart weight (HW) to tibia length (TL) HW:TL of mice, **(C)** Cytosolic phosphorylated Akt and total Akt protein expression in young and aged WT hearts and sEH null hearts. Values represent mean ± SEM, *n* = 5–8, *P* < 0.05, ^∗^vs. young counterpart; ‡vs female counterpart.

Significant increases in body weight were observed in all aged mice of both sexes and genotypes (Figure 1A). The ratio of heart weight (HW) to tibia length (TL) was used as an index of cardiac hypertrophy. There were no differences in cardiac weights between young WT and sEH null animals of either sex (Figure 1B). Both aged male and female WT mice and male sEH null mice demonstrated significant increases in HW:TL; however, no increases were observed in aged female sEH null mice (Figure 1B). While the Akt pathway has an important role as a pro-survival pathway, increased activation of Akt over aging has been shown to contribute to age-related cardiac hypertrophy and inflammation (Hua et al., 2011; Chen et al., 2017). Consistent with the literature, immunoblotting results indicated significantly increased levels of pAkt in the cytosolic fraction in aged mice (Figure 1C).

### sEH Genetic Deletion Preserves Cardiac Function in Aged Mice

Characterization of aged and sex-related changes to cardiac function and parameters were obtained using 2D echocardiography. Results demonstrated there were no significant differences in heart rate between any of the groups. Yet, ejection fraction (% EF) and fractional shortening (% FS), markers of systolic function, significantly decreased in aged male and female WT mice as well as sEH null males (Table 1). Similarly, left ventricular end diastolic (LVEDV) and end systolic (LVESV) volumes and chamber internal diameters (LVID) increased in aged male and female WT mice and aged sEH null males, indicating a general decline in systolic function (Table 1). Interestingly, aged female sEH null mice were not different from young mice, indicating a general preservation of systolic function (Table 1). There were no significant changes in isovolumetric contraction or relaxation times (IVCT or IVRT) between any groups, but aortic ejection time (AET) was preserved only in aged male sEH null mice. The E/A ratio is a measure of mitral blood flow and is commonly used as a marker of diastolic function. E/A was significantly decreased in both WT male and female aged mice. Interestingly, the E/A ratio was significantly decreased in aged female but not male sEH null mice (Table 1). Similarly, E/E’, a marker of LV filling pressure, was significantly decreased in female but not male mice. Together, these data suggest a divergent effect of sEH genetic deletion, with aged female mice exhibiting preserved systolic function and aged males exhibiting preserved diastolic function.

**TABLE 1:** Cardiac functional parameters measured by 2D echocardiography.

	WT				sEH null			
	Female		Male		Female		Male	
	Young	Aged	Young	Aged	Young	Aged	Young	Aged
HR, beats/min	460 ± 12	466 ± 7	459 ± 11	467 ± 9	461 ± 8	494 ± 9	479 ± 13	457 ± 15
**Wall measurements**								
EF, %	71.43 ± 1.90	61.62 ± 1.34*	69.11 ± 2.03	57.95 ± 1.22*	69.64 ± 1.66	65.15 ± 1.65	68.53 ± 1.49	57.41 ± 1.91*‡
FS, %	40.53 ± 1.69	32.86 ± 0.93*	38.63 ± 1.67	30.55 ± 0.83*	38.85 ± 1.29	35.49 ± 1.21	37.94 ± 1.14	30.12 ± 1.27*
LVEDV, μl	55.34 ± 2.83	72.04 ± 3.79*	72.34 ± 5.85‡	90.62 ± 2.35*‡	59.28 ± 2.51	69.27 ± 3.41	62.92 ± 2.90	81.75 ± 4.39*
LVESV, μl	16.97 ± 1.61	28.29 ± 2.26*	23.30 ± 2.86	37.63 ± 1.55*‡	18.89 ± 1.64	24.28 ± 2.19	20.12 ± 1.46	35.69 ± 3.18*‡
CO, ml/min	17.78 ± 0.96	20.39 ± 1.01	22.13 ± 1.64	24.74 ± 0.87	18.66 ± 0.79	22.15 ± 0.86	19.90 ± 1.06	20.81 ± 0.68‡
SV, μl	38.37 ± 1.52	43.75 ± 1.82	49.03 ± 3.35‡	52.99 ± 1.58‡	40.41 ± 1.36	44.99 ± 1.59	42.80 ± 2.06	46.07 ± 1.85
Corrected LV mass, mg	66.83 ± 3.60	107.65 ± 6.33*	90.88 ± 7.71‡	142.51 ± 4.57*‡	78.40 ± 3.01	99.73 ± 3.47*	83.25 ± 3.05	117.38 ± 5.20*#
LA, mm	1.59 ± 0.08	2.06 ± 0.12*	1.52 ± 0.09	2.17 ± 0.10*	1.50 ± 0.07	1.98 ± 0.08*	1.59 ± 0.08	2.14 ± 0.06*
IVS-diastole, mm	0.69 ± 0.03	0.86 ± 0.03*	0.73 ± 0.03	0.85 ± 0.02	0.77 ± 0.02	0.85 ± 0.02	0.77 ± 0.03	0.74 ± 0.03
IVS-systole, mm	1.13 ± 0.05	1.26 ± 0.05	1.19 ± 0.05	1.39 ± 0.03*	1.16 ± 0.04	1.27 ± 0.04	1.15 ± 0.04	1.20 ± 0.03^#^
LVPW-diastole, mm	0.71 ± 0.03	0.88 ± 0.03*	0.82 ± 0.04	0.96 ± 0.03*	0.77 ± 0.03	0.86 ± 0.02	0.82 ± 0.03	0.90 ± 0.03
LVPW-systole, mm	1.13 ± 0.04	1.27 ± 0.04	1.28 ± 0.06	1.34 ± 0.03	1.16 ± 0.04	1.25 ± 0.03	1.25 ± 0.04	1.20 ± 0.04
LVID-diastole, mm	3.60 ± 0.08	4.02 ± 0.09*	3.96 ± 0.14	4.45 ± 0.05*‡	3.69 ± 0.07	3.94 ± 0.08	3.75 ± 0.10	4.25 ± 0.10*
LVID-systole, mm	2.16 ± 0.10	2.71 ± 0.09*	2.44 ± 0.13	3.07 ± 0.05*‡	2.27 ± 0.08	2.55 ± 0.09	2.34 ± 0.08	2.98 ± 0.12*‡
**Doppler Imaging**								
IVRT, ms	17.14 ± 0.97	16.83 ± 0.67	16.67 ± 1.64	15.62 ± 0.47	15.16 ± 0.60	15.06 ± 0.83	15.18 ± 0.78	15.61 ± 0.55
IVCT, ms	12.99 ± 1.22	14.98 ± 0.92	15.00 ± 1.18	16.17 ± 1.03	13.61 ± 1.18	11.98 ± 0.84	12.30 ± 0.72	15.70 ± 0.69
AET, ms	51.70 ± 1.77	42.30 ± 1.03*	48.91 ± 2.40	41.77 ± 0.95*	50.07 ± 1.47	40.03 ± 0.81*	46.02 ± 1.49	43.12 ± 1.31
Tei index	0.581 ± 0.025	0.758 ± 0.029*	0.663 ± 0.049	0.766 ± 0.024	0.574 ± 0.022	0.678 ± 0.034	0.604 ± 0.024	0.727 ± 0.020
E/A	1.88 ± 0.06	1.55 ± 0.06*	1.75 ± 0.09	1.44 ± 0.05*	1.68 ± 0.06	1.43 ± 0.05*	1.63 ± 0.03	1.50 ± 0.03
E′/A′	1.32 ± 0.07	1.22 ± 0.05	1.27 ± 0.09	1.17 ± 0.07	1.39 ± 0.12	1.25 ± 0.11	1.21 ± 0.05	1.09 ± 0.07
E′	22.40 ± 1.44	23.21 ± 1.05	24.95 ± 1.58	22.16 ± 1.17	22.01 ± 1.29	25.55 ± 1.51	23.49 ± 1.75	20.72 ± 1.47
E/E′	30.48 ± 2.06	22.70 ± 1.14*	29.98 ± 2.22	25.43 ± 1.50	29.87 ± 1.43	26.41 ± 1.04	31.03 ± 2.22	32.29 ± 2.35
*n* number	20	24	13	31	20	21	20	17

Data is shown as mean ± SEM, *P* < 0.05, * vs. young counterpart; #vs, WT counterpart; ‡vs, female counterpart.

### Aging Affects the Protein Expression of Epoxide Hydrolases

**FIGURE 2:**
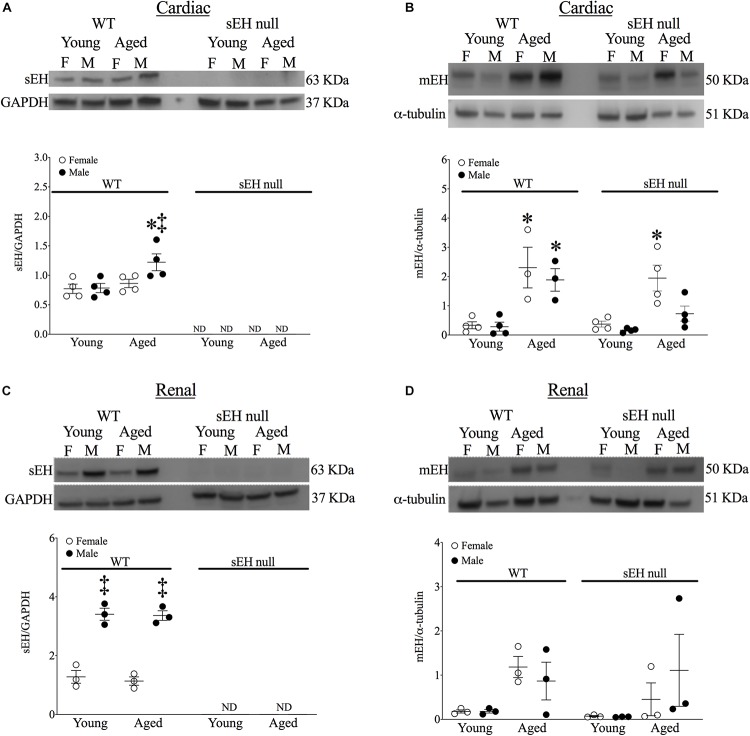
Protein expression of epoxide hydrolases in WT and sEH null mice. Representative immunoblots and quantitation for cardiac soluble epoxide hydrolase (sEH, **A**) and microsomal epoxide hydrolase (mEH, **B**). Representative immunoblots for renal sEH **(C)** and mEH **(D)**. Protein expression of sEH was normalized to GAPDH. Protein expression of mEH was normalized to α-tubulin. Data represented as mean ± SEM, *n* = 4–5, *P* < 0.05, ^∗^*vs. young counterpart; ‡vs. female group.

No expression of sEH was detected in either young or aged hearts from null mice confirming genetic deletion (Figure 2A). sEH expression was significantly increased in aged male WT mice but not in aged females (Figure 2A). Interestingly, our data demonstrated a significant increase in mEH expression in both aged WT and sEH null females, as well as aged WT males (Figure 2B). However, this increase was not observed in aged sEH null males, who had significantly decreased mEH expression compared to aged WT males (Figure 2B). Epoxide hydrolases have an important role in renal epoxylipid metabolism; as renal function is important to overall cardiovascular health, we assessed changes in sEH and mEH in kidneys (Imig, 2006). There was no expression of sEH observed in kidneys from sEH null mice (Figure 2C). Correlating with previous literature, renal sEH expression was significantly increased in WT males compared to females but there were no differences between young and aged mice (Figure 2C; Sinal et al., 2000). Renal mEH was not significantly altered in any group (Figure 2D).

### Sirt-3 Activity and Acetylated MnSOD Are Preserved in Aged sEH Null Female Mice

**FIGURE 3:**
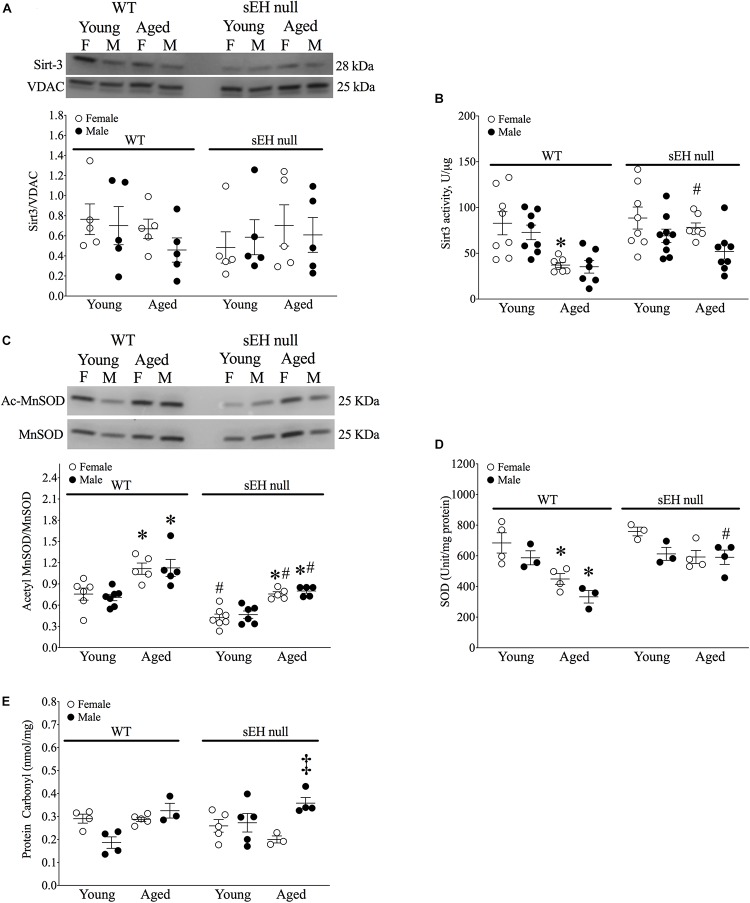
Sirtuin-3 and SOD in young and aged WT and sEH null mice. **(A)** Cardiac Sirt-3 protein expression in young and aged WT and sEH null mice. **(B)** Sirt-3 activity in young and aged WT and sEH null mice was determined in mitochondrial fractions. **(C)** Relative protein expression of AcMnSOD normalized to total MnSOD in young and aged WT hearts and sEH null hearts. **(D)** Superoxide dismutase (SOD) activity was assessed in cytosolic fractions from young and aged WT and sEH null hearts. **(E)** Protein carbonylation levels were assessed in cardiac cytosolic fractions from young and aged WT and sEH null hearts. Data represented as mean ± SEM, *n* = 4–8, *P* < 0.05, ^∗^vs young counterpart; #vs WT counterpart; ‡vs female counterpart.

**FIGURE 4:**
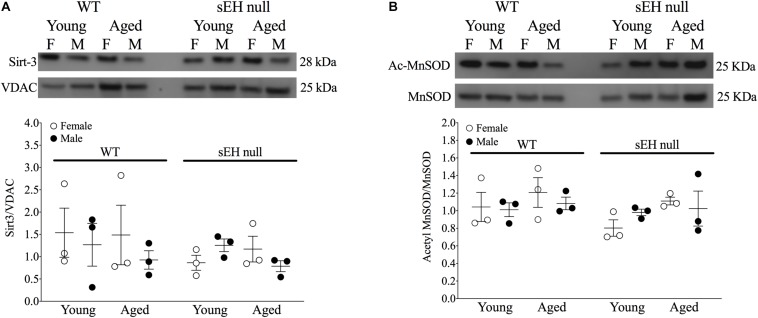
Renal expression of mitochondrial Sirt-3 compared to VDAC **(A)** and Acetylated MnSOD **(B)** compared to total MnSOD. Data represented as mean ± SEM, *n* = 3, *P* < 0.05.

Sirt-3, the main mitochondrial deacetylase, has been found to be down-regulated with aging and associated with increased ROS levels correlating with a decline in cardiac function (Brown et al., 2013; Zeng et al., 2014). In the current study, there were no differences observed in mitochondrial Sirt-3 protein expression in any group (Figure 3A). However, Sirt-3 activity was significantly decreased in hearts from aged WT females, with a similar trend in males (*P* = 0.0726). Interestingly, Sirt-3 activity was preserved in aged sEH null mice compared to their young counterparts and aged sEH null females had significantly higher Sirt-3 activity than similarly aged WT females (Figure 3B). The age-dependent changes in Sirt-3 result in reduction in the level of activated MnSOD resulting in increased oxidative stress (Hua et al., 2011). Consistent with previous studies, the expression level of AcMnSOD significantly increased in an age-dependent manner in both male and female WT hearts (Figure 3C). Interestingly, AcMnSOD expression was lower in sEH null mice compared to their WT counterparts (Figure 3C). Both aged male and female sEH null mice demonstrated increased AcMnSOD levels but these were significantly lower than the corresponding aged WT mice (Figure 3C). In accordance with literature, cardiac SOD activity was significantly decreased in both male and female WT aged animals (Figure 3D). SOD activity was preserved in aged sEH null animals compared to the young null mice (Figure 3D). These data suggest sEH genetic deletion confers protection against oxidative stress by preserving Sirt3 activity, which will decrease AcMnSOD levels resulting in maintained SOD activity. This was partially supported by assessment of protein carbonylation as a biomarker of oxidative stress (Fedorova et al., 2014), where increased levels of protein carbonyl were only observed in aged male animals regardless of their genotype and not in aged female hearts (Figure 3E). Renal AcMnSOD and Sirt-3 expression remained unchanged in all groups with age (Figures 4A,B).

### Cardiac Mitochondrial Ultrastructure Is Preserved in Aged Female sEH Null Mice

**FIGURE 5:**
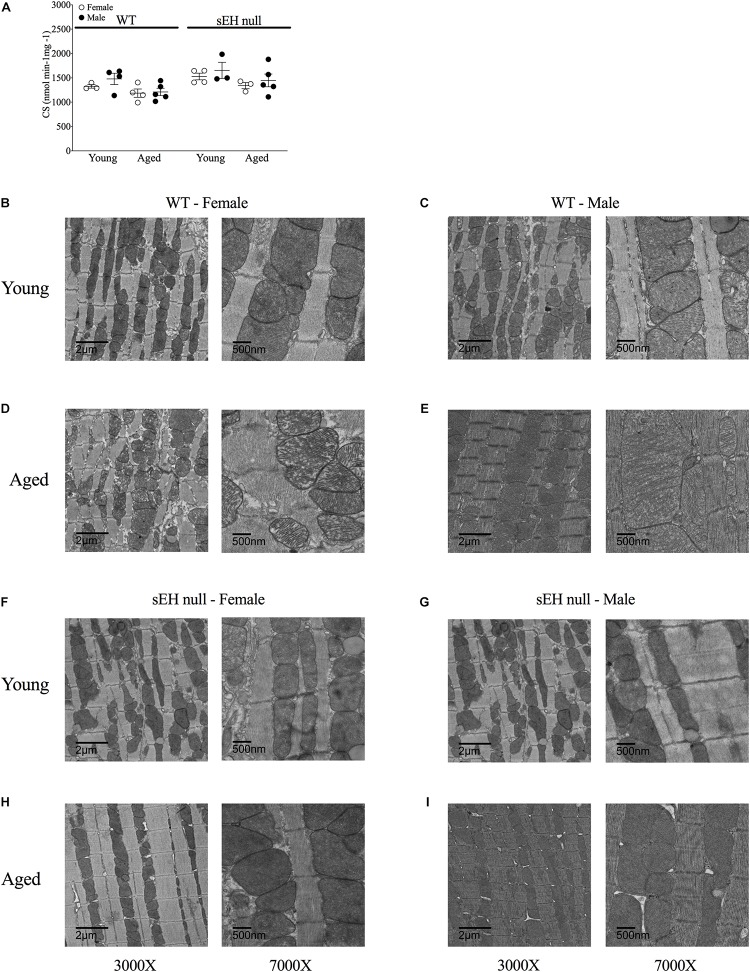
Mitochondrial content and ultrastructure in hearts from young and aged WT and sEH null mice. **(A)** Citrate synthase activity level as a biomarker of mitochondrial content in young and aged WT and sEH null mice was determined spectrophotometrically, data are represented as mean ± SEM, *n* = 5–8. Representative transmission electron micrograph images from WT **(B–E)** and sEH null **(F–I)** mice at two magnifications, 3000X (left) and 7000X (right), *n* = 1 per group.

To obtain an estimate of cardiac mitochondrial content, we measured the activity of citrate synthase, a rate-limiting enzyme involved in mitochondrial oxidative metabolism (Larsen et al., 2012). There were no differences in citrate synthase activity in any group suggesting the overall mitochondrial content was not significantly altered (Figure 5A). Conventional TEM was employed to assess mitochondrial ultrastructure in the left ventricular free wall of both young and aged mice. Marked alterations in mitochondrial ultrastructure, exemplified by decreased cristae density, disturbed arrangement in the myofibrillar spaces and enlarged size, were observed in both male and female aged WT hearts (Figures 5B–E). The age-related changes to mitochondrial morphology were absent in sEH null animals (Figures 5F–I).

### Age Is a Crucial Variable in Cardiac Function and Oxidative Stress Responses

Variance (η^2^) from three-way ANOVA (age x genotype x sex) and resultant significance are listed in Tables 2, 3, respectively. Of the three variables assessed in this study, age exhibited the greatest significant effect over the majority of parameters assessed (Tables 2, 3). Sex was also a significant contributor to the effects observed in cardiac systolic function, Sirt-3 activity and epoxide hydrolase expression (Tables 2, 3). Genotype demonstrated significant effects on heart weight, cardiac Sirt-3 activity, and SOD activity and diastolic cardiac function (Tables 2, 3). Interaction effects delineate the effect of one variable, such as age, on the impact of another, such as genotype, for any parameter in question. When an interaction is significant for a given parameter, it suggests the impact depends on the presence of the other factor. Important interaction effects between age, genotype and sex are described in Tables 2, 3. There was no interaction between all three variables for any factor (data not shown). These data support the evidence demonstrating the importance of age and sex in cardiac function.

**TABLE 2:** Proportional variance (η^2^) values for age, genotype and sex as assessed by three-way ANOVA.

Variable	Age	Genotype	Sex	Age × Genotype	Age × Sex	Genotype × Sex
**Physiological parameters**						
Body weight (g)	**0.4993**	0.0011	**0.0732**	0.0005	0.0010	0.0041
HW:TL, mg/mm	**0.2825**	**0.0233**	**0.1745**	**0.0332**	**0.0258**	ns
HR, beats/min	0.0040	0.0105	0.0020	ns	0.0191	0.0021
**Protein expression**						
pAKT/AKT	**0.5828**	0.0029	0.0355	0.0253	0.0046	0.0022
Cardiac sEH	**0.0185**	**0.8821**	**0.0092**	**0.0185**	0.0080	**0.0092**
Cardiac mEH	**0.5598**	0.0437	**0.0617**	0.0352	0.0326	0.0157
Renal sEH	0.0003	**0.6771**	**0.1524**	0.0003	0.0001	**0.1524**
Renal mEH	**0.3350**	0.0178	0.0036	0.0022	0.0043	0.0312
Cardiac Sirt-3	0.0012	0.0060	0.0096	0.0457	0.0160	0.0108
Cardiac AcMnSOD	**0.4116**	**0.3121**	0.0004	0.0023	0.0007	0.0029
Renal Sirt-3	0.0133	0.0565	0.0293	0.0023	0.0491	0.0304
Renal AcMnSOD	0.1294	0.0681	0.0015	0.0049	0.0478	0.0231
**Enzymatic activity**						
Cardiac Sirt-3, U ⋅ μg^–1^	**0.2208**	**0.0643**	**0.0598**	**0.0562**	ns	0.0209
SOD, U ⋅ mg^–1^	**0.3586**	**0.1958**	**0.1017**	**0.0708**	0.0123	0.0033
Protein Carbonylation, nmol ⋅ mg^–1^	**0.0800**	ns	0.0337	0.0365	**0.2499**	**0.1725**
Citrate Synthase, nmol ⋅ min^–1^mg^–1^	**0.1827**	**0.1680**	0.0481	0.0001	0.0063	0.0009
**2D ECHO: Wall measurements**						
EF,%	**0.2549**	0.0001	**0.0420**	0.0055	0.0121	0.0016
FS,%	**0.2476**	ns	**0.0374**	0.0071	0.0081	0.0015
LVEDV, μl	**0.1663**	0.0119	**0.1092**	0.0016	0.0045	**0.0155**
LVESV, μl	**0.2293**	0.0055	**0.0846**	0.0023	**0.0183**	0.0010
CO, ml/min	**0.0575**	0.0078	**0.0460**	0.0004	0.0042	**0.0481**
SV, μl	**0.0492**	0.0163	**0.0910**	0.0004	0.0012	**0.0449**
Corrected LV mass, mg	**0.3063**	**0.0119**	**0.0928**	**0.0192**	0.0078	**0.0186**
LA, mm	**0.3451**	0.0011	0.0064	0.0005	0.0041	0.0032
IVS-diastole, mm	**0.1061**	0.0000	0.0057	**0.0521**	**0.0278**	**0.0245**
IVS-systole, mm	**0.0800**	0.0122	0.0043	0.0099	0.0000	**0.0281**
LVPW-diastole, mm	**0.1490**	0.0004	**0.0493**	0.0130	0.0006	0.0071
LVPW-systole, mm	**0.0249**	0.0109	**0.0283**	0.0101	0.0184	0.0140
LVID-diastole, mm	**0.1769**	0.0099	**0.0866**	0.0012	0.0059	0.0108
LVID-systole, mm	**0.2497**	0.0034	**0.0737**	0.0033	0.0112	0.0013
**2D ECHO: Doppler imaging**						
IVRT, ms	0.0011	**0.0299**	0.0013	0.0030	ns	0.0055
IVCT, ms	0.0140	0.0176	0.0181	0.0011	0.0101	0.0004
AET, ms	**0.2371**	0.0081	0.0050	0.0035	**0.0241**	0.0015
Tei index	**0.1613**	**0.0214**	0.0179	0.0017	0.0020	0.0001
E/A	**0.1864**	**0.0246**	0.0082	0.0103	0.0042	0.0117
E′/A′	0.0237	0.0002	0.0207	0.0003	0.0000	0.0066
E′	0.0006	0.0004	0.0014	0.0030	**0.0394**	0.0095
E/E′	**0.0494**	**0.0284**	0.0201	**0.0241**	0.0148	0.0054

*Variances are reported as a fraction of 1. Bolded values represent variances that reach a statistically significant contribution of the total variability, *P* < 0.05.*

**TABLE 3:** *P*-values from three-way ANOVA statistical analysis of data-set variability.

Variable	Age	Genotype	Sex	Age × Genotype	Age × Sex	Genotype × Sex
**Physiological parameters**						
Body weight (g)	**<0.0001**	0.4404	**<0.0001**	0.5936	0.4583	0.1366
HW:TL, mg/mm	**<0.0001**	**0.0474**	**<0.0001**	**0.0190**	**0.0374**	0.9715
HR, beats/min	0.4141	0.1860	0.5623	0.9422	0.0755	0.5523
**Protein expression**						
pAKT/AKT	**<0.0001**	0.5743	0.0556	0.1034	0.4802	0.6218
Cardiac sEH	**0.0043**	**<0.0001**	**0.0371**	**0.0043**	0.0513	**0.0371**
Cardiac mEH	**<0.0001**	0.0771	**0.0384**	0.1103	0.1235	0.2783
Renal sEH	0.6159	**<0.0001**	**<0.0001**	0.6159	0.7943	**<0.0001**
Renal mEH	**0.0075**	0.4902	0.7553	0.8066	0.7332	0.3642
Cardiac Sirt-3	0.8365	0.6480	0.5653	0.2143	0.4585	0.5423
Cardiac AcMnSOD	**<0.0001**	**<0.0001**	0.8027	0.5654	0.7577	0.5196
Renal Sirt-3	0.6153	0.3061	0.4578	0.8346	0.3391	0.4496
Renal AcMnSOD	0.1080	0.2347	0.8553	0.7437	0.3160	0.4819
**Enzymatic activity**						
Cardiac Sirt-3, U ⋅μg^–1^	**<0.0001**	**0.0187**	**0.0231**	**0.0275**	0.9616	0.1730
SOD, U ⋅ mg^–1^	**<0.0001**	**0.0013**	**0.0140**	**0.0360**	0.3605	0.6308
Protein carbonylation, nmol ⋅ mg^–1^	**0.0496**	0.9944	0.1923	0.1753	**0.0012**	**0.0056**
Citrate synthase, nmol ⋅ min^–1^mg^–1^	**0.0133**	**0.0170**	0.1819	0.9639	0.6240	0.8539
**2D ECHO: Wall measurements**						
EF,%	**<0.0001**	0.8915	**0.0017**	0.2520	0.0885	0.5388
FS,%	**<0.0001**	0.9607	**0.0034**	0.1970	0.1686	0.5572
LVEDV, μl	**<0.0001**	0.0836	**<0.0001**	0.5300	0.2893	**0.0491**
LVESV, μl	**<0.0001**	0.2324	**<0.0001**	0.4358	**0.0295**	0.6135
CO, ml/min	**0.0010**	0.2195	**0.0031**	0.7773	0.3677	**0.0025**
SV, μl	**0.0017**	0.0674	**<0.0001**	0.7803	0.6119	**0.0026**
Corrected LV mass, mg	**<0.0001**	**0.0379**	**<0.0001**	**0.0087**	0.0919	**0.0098**
LA, mm	**<0.0001**	0.6172	0.2364	0.7435	0.3450	0.4060
IVS-diastole, mm	**<0.0001**	0.9723	0.3089	**0.0024**	**0.0252**	**0.0357**
IVS-systole, mm	**0.0001**	0.1253	0.3639	0.1670	0.9575	**0.0208**
LVPW-diastole, mm	**<0.0001**	0.7640	**0.0014**	0.0969	0.7176	0.2189
LVPW-systole, mm	**0.0351**	0.1611	**0.0250**	0.1785	0.0694	0.1128
LVID-diastole, mm	**<0.0001**	0.1205	**<0.0001**	0.5948	0.2308	0.1058
LVID-systole, mm	**<0.0001**	0.3423	**<0.0001**	0.3524	0.0875	0.5627
**2D ECHO: Doppler imaging**						
IVRT, ms	0.6663	**0.0276**	0.6412	0.4789	0.9306	0.3423
IVCT, ms	0.1210	0.0825	0.0785	0.6611	0.1865	0.8040
AET, ms	**<0.0001**	0.1804	0.2914	0.3758	**0.0217**	0.5603
Tei index	**<0.0001**	**0.0379**	0.0573	0.5511	0.5252	0.9008
E/A	**<0.0001**	**0.0233**	0.1893	0.1401	0.3476	0.1164
E′/A′	0.0540	0.8518	0.0713	0.8288	0.9445	0.3068
E′	0.7670	0.8147	0.6478	0.4998	**0.0155**	0.2318
E/E′	**0.0045**	**0.0301**	0.0670	**0.0455**	0.1154	0.3413

*Bolded values represent variables that elicit statistically significant effects on the parameter in question, *P* < 0.05.*

## DISCUSSION

Biological aging is a natural process resulting in marked changes to an individual’s ability to overcome stress, which worsen adverse outcomes such as increased CVD risk (Paneni et al., 2017). Although the underlying mechanisms behind cardiac aging remain elusive, mitochondrial dysfunction is hypothesized to be a major contributor (Chaudhary et al., 2011). In particular, age-associated mitochondrial damage leading to increased ROS production damaging mitochondrial DNA and proteins as well affecting quality control processes, ultimately contributing to decreased cardiac function (Poljsak and Milisav, 2013; Chiao and Rabinovitch, 2015; Steenman and Lande, 2017). Importantly, sex and gender differences are known to affect the etiology, presentation and prognosis of CVDs as individuals age (Shaw et al., 2009; Parker et al., 2010; Wenger, 2012). Unique CVD risks for women include the cessation of menarche (menopause), preeclampsia, gestational diabetes and certain autoimmune inflammatory disorders, such as systemic lupus erythematosus (Aggarwal et al., 2018). While men on average present with CVD at a younger age, evidence suggests CVD risk factors such as hypertension and diabetes play a greater role in disease acceleration in women (Cheng et al., 2010). A further understanding and characterizing potential sex-specific mechanisms contributing to cardiac aging may provide new insights for the optimal prevention and management of age-related CVDs. Consistent with literature, we report sex-differences in the age-associated development of myocardial hypertrophy and deterioration of cardiac function; however, we demonstrate novel data highlighting the beneficial effect of deleting sEH.

In humans, sex-dependent differences in cardiac aging indicate males on average exhibit greater impaired systolic function coupled with increased wall thickness, cavity dimension and LV mass (Merz and Cheng, 2016). Conversely women display a greater degree of diastolic impairment coupled with increased concentric remodeling, with systolic impairment occurring later than in their male counterparts (Krumholz et al., 1993; Cheng et al., 2010). Murine models are unable to replicate changes in blood pressure and blood cholesterol often present in human patients with CVD; however, they are a useful model recapitulating many human age-related changes in cardiac structure and function, such as increased LV mass, decreased diastolic filling ratios and reduced fractional shortening (Dai and Rabinovitch, 2009). In the present study, we observed a significant decline in systolic and diastolic parameters coupled with a significant increase in LV mass in aged male and female WT mice. These data are consistent with what is observed clinically in aging humans. Interestingly, aged female sEH null mice demonstrated preserved systolic function and LV mass but exhibited diastolic dysfunction. Conversely, aged male sEH null mice demonstrated a significant reduction in systolic function but no significant change in diastolic parameters. Previously, we demonstrated cardioprotective effects in aged sEH null mice following myocardial infarction (Jamieson et al., 2017b). This previous study used combined males and females and was not designed to assess sex differences, but rather generalized aging effects in an injury model. The present data suggest important sex-specific differences in cardiac aging following sEH genetic deletion in the absence of any defined disease state.

Sexual dimorphism in sEH expression and activity has been documented in the renal, hepatic and cardiovascular systems in young rodent models (Pinot et al., 1995; Sinal et al., 2000; Zhang et al., 2013; Qin et al., 2016; Yang et al., 2018), yet the exact mechanism(s) behind these differences remain unknown. Recent studies have demonstrated estrogen/estrogen receptor mediated methylation of the sEH promoter region causes gene silencing in female rodents (Yang et al., 2018). This epigenetic silencing of sEH expression may be responsible for some sexual dimorphism observed in young animal models, although whether this occurs in aged animals is unknown. In the current study, we observed an increase in sEH expression in aged WT males that was absent in WT females. In addition, there was an age-related increase in mEH expression in WT mice and sEH null females, but not sEH null males. The increased mEH expression in females may be a compensatory response to the sEH deletion but it is unknown why this does not occur in aged sEH null males. The role of mEH in cardiac eicosanoid metabolism has only recently been investigated *in vivo*. Early data suggested sEH demonstrated a higher catalytic ability compared to mEH and played the predominate role in epoxylipid metabolism (Spector and Norris, 2007; Marowsky et al., 2009; Harris and Hammock, 2013). In contrast, mEH had been considered an important mediator of xenobiotic metabolism, with limited contribution to cardiac epoxylipid metabolism (Marowsky et al., 2009). Recent data from Edin et al. suggested under basal conditions it is substrate availability rather than catalytic activity that drives epoxylipid metabolism (Edin et al., 2018). Cell injury caused by stressors such as ischemia can promote the release of free arachidonic acid, where sEH plays the dominate role in epoxylipid metabolism. Conversely, under basal or physiological conditions mEH may act as a “first-pass” hydrolase to remove the small amount of endogenous epoxylipids produced (Edin et al., 2018). Furthermore, they suggest while tethering of mEH to the microsomes may hinder its ability to scavenge epoxylipids from the cytosol, epoxylipids bound in the microsomes will be in close proximity. Interestingly, the current study demonstrates both mEH and sEH significantly increased in aged WT males but only mEH increased in aged WT and sEH null females. Whether these differences with age and sex are related to changes in epoxylipid formation, shifts in epoxylipid storage or alterations in enzymatic catalytic activity remain unknown and are the subject of ongoing research. Interestingly, female sEH null mice were protected against aged-dependent development of hypertrophy and had preserved cardiac systolic function, while aged sEH null male mice were not protected against hypertrophy but demonstrated preserved diastolic function.

Mitochondria are powerful organelles essential for maintaining cardiac function through oxidative phosphorylation and ATP generation; however, they are also the main site of ROS production (Chen and Zweier, 2014; Siasos et al., 2018). During the aging process in both mice and humans cardiac ROS production outpaces mitochondrial scavenging capacity correlating with the decline in function (Panth et al., 2016; Brown et al., 2017). Evidence of sex specific differences in mitochondrial function and morphology have been observed in healthy and diseased states but the underlying molecular mechanisms remain poorly understood (Justo et al., 2005). For example, cardiomyocytes from female rats have been found to possess lower mitochondrial content yet exhibit more efficient mitochondria compared to males (Colom et al., 2007). Furthermore, female rats show lower levels of mitochondrial hydrogen peroxide in liver and brain (Borrás et al., 2003). In the current study, an age-related increase in the level of protein carbonylation was observed in males indicating a significant increase in cardiac oxidative stress. MnSOD is the primary mitochondrial antioxidant enzyme that contributes to maintaining mitochondrial function; moreover, inactivation of the MnSOD gene in mice results in neonatal lethality (Li et al., 1995; Brown et al., 2007). In rat brain and liver, higher expression and activity of MnSOD in females is associated with lower oxidative damage (Borrás et al., 2003). The activation of MnSOD is primarily regulated through its deacetylation via Sirt-3, which is the predominant mitochondrial deacetylase (Parodi-Rullán et al., 2018). Sirt-3 deficient mice demonstrate mitochondrial dysfunction and excessive production of ROS as well cardiac fibrosis and hypertrophy (Sundaresan et al., 2016; Wei et al., 2017). It has been reported that the hyperacetylation and deactivation of mitochondrial proteins including MnSOD over aging is associated with a decline in Sirt-3 activity (Parodi-Rullán et al., 2018). The decline in Sirt-3 activity coupled with a significant increase in expression of AcMnSOD in aged WT animals is consistent with the literature. Importantly, our data demonstrated sEH deletion preserved Sirt-3 activity in aged mice and was associated with reduced expression of AcMnSOD. Moreover, SOD activity was reduced significantly in WT mice but not in sEH null mice. Importantly, the increased antioxidant activity of mitochondrial SOD observed in sEH null mice correlated with better mitochondrial ultrastructure. Recent evidence suggests Sirt-3 potentially has a role in limiting cardiac hypertrophy as it is found to be downregulated in mouse hypertrophic hearts (Chen et al., 2015; Koentges et al., 2016). Sirt-3 mediated activation of MnSOD and subsequent ROS scavenging is proposed to suppress hypertrophic signaling, such as the PI3K/Akt pathway (Pillai et al., 2014). Interestingly, the increase in cardiac pAkt expression observed in all aged mice did not correlate with the oxidative stress or hypertrophic responses observed in the aged mice. Thus, these data suggest the genetic deletion of sEH provided a better capacity for cardiac mitochondria to limit potential aged-related damage, which was independent of an Akt pathway.

Many of the protective effects attributed to sEH gene deletion have been associated with increased epoxylipid levels, such as increased levels of epoxysatrienoic acids (EETs). Peroxisome proliferator-activated receptor gamma co-activator 1-alpha (PGC-1α) is known to mediate mitochondrial function, oxidative stress and Sirt-3 expression (Kong et al., 2010). In models of obesity, EETs have been shown to activate PGC-1α, resulting in preserved mitochondrial structural and functional proteins associated with preserved Sirt-3 expression (Singh et al., 2016). In the present study, while we observed no change in Sirt-3 expression, we observed a preservation of Sirt-3 activity. These data suggest there may be post-translational modification(s) of Sirt-3 associated with sEH genetic deletion and subsequent altered epoxylipid metabolism that are independent to the effects mediated through PGC-1α. Pillai et al. reported the biophenolic compound hokoniol is capable of passing through mitochondrial membranes to directly bind Sirt-3, improving affinity of Sirt-3 binding and utilization of NAD^+^, ultimately preserving its deacetylase activity (Singh et al., 2016; Ansari et al., 2017). Preservation of Sirt-3 activity observed in sEH null mice has not previously been reported. While the mechanisms responsible for the age-dependent preservation of Sirt-3 activity observed in sEH null mice is unknown, we propose a resulting increase in epoxylipid metabolites have a role in conserving the deacetylase activity of Sirt-3 over aging.

In the current study, we characterize the effect and sexual dimorphisms of sEH deletion in cardiac aging. The data demonstrated aged sEH null mice have preserved Sirt-3 activity, decreased AcMnSOD levels and better mitochondrial ultrastructure compared to WT mice. Interestingly, sEH null females had preserved systolic function and no cardiac hypertrophy, while sEH null male mice had preserved diastolic function. Increased expression of sEH was observed in WT males and marked increases in mEH expression where found in both genotypes. While further studies are necessary to elucidate the mechanism(s) behind these effects, the data highlight novel sexual dimorphic patterns of cardiac aging.

## Data Availability Statement

The datasets generated for this study are available on request to the corresponding author.

## Ethics Statement

The animal study was reviewed and approved by the Research Ethics, Animal Care and Use Committees Health Sciences, University of Alberta.

## Author Contributions

KLJ and HK-B were equal contributors involved in study design, data acquisition and analysis, and writing of the manuscript. AD and DS performed the key individual experiments and aided in the writing of the manuscript. JS was the PI and involved in study design, data analyses, and writing of the manuscript.

## Conflict of Interest

The authors declare that the research was conducted in the absence of any commercial or financial relationships that could be construed as a potential conflict of interest.

## References

[B1] Aggarwal N. R. Patel H. N. Mehta L. S. Sanghani R. M. Lundberg G. P. Lewis S. J. (2018). Sex differences in ischemic heart disease: advances, obstacles, and next steps. *Circ. Cardiovasc. Qual. Outcomes* 11:e004437. 10.1161/CIRCOUTCOMES.117.004437 29449443

[B2] Ai D. Pang W. Li N. Xu M. Jones P. D. Yang J. (2009). Soluble epoxide hydrolase plays an essential role in angiotensin II-induced cardiac hypertrophy. *Proc. Natl. Acad. Sci. U.S.A.* 106 564–569. 10.1073/pnas.0811022106 19126686 PMC2626743

[B3] Akhnokh M. K. Yang F. H. Samokhvalov V. Jamieson K. L. Cho W. J. Wagg C. (2016). Inhibition of soluble epoxide hydrolase limits mitochondrial damage and preserves function following ischemic injury. *Front. Pharmacol.* 7:133. 10.3389/fphar.2016.00133 27375480 PMC4896112

[B4] Ansari A. Rahman M. S. Saha S. K. Saikot F. K. Deep A. Kim K. H. (2017). Function of the SIRT3 mitochondrial deacetylase in cellular physiology, cancer, and neurodegenerative disease. *Aging Cell* 16 4–16. 10.1111/acel.12538 27686535 PMC5242307

[B5] Benigni A. Perico L. Macconi D. (2016). Mitochondrial dynamics is linked to longevity and protects from end-organ injury: the emerging role of sirtuin 3. *Antioxid. Redox Signal.* 25 185–199. 10.1089/ars.2016.6682 26972664

[B6] Benjamin E. J. Muntner P. Bittencourt M. S. (2019). Heart disease and stroke statistics-2019 update: a report from the American Heart Association. *Circulation* 139 e56–e528. 30700139 10.1161/CIR.0000000000000659

[B7] Beyer W. F. Jr. Fridovich I. (1987). Assaying for superoxide dismutase activity: some large consequences of minor changes in conditions. *Anal. Biochem.* 161 559–566. 10.1016/0003-2697(87)90489-1 3034103

[B8] Bochaton T. Crola-Da-Silva C. Pillot B. Villedieu C. Ferreras L. Alam M. R. (2015). Inhibition of myocardial reperfusion injury by ischemic postconditioning requires sirtuin 3-mediated deacetylation of cyclophilin D. *J. Mol. Cell. Cardiol.* 84 61–69. 10.1016/j.yjmcc.2015.03.017 25871830

[B9] Borrás C. Sastre J. García-Sala D. Lloret A. Pallard F. V. Viña J. (2003). Mitochondria from females exhibit higher antioxidant gene expression and lower oxidative damage than males. *Free Radic. Biol. Med.* 34 546–552. 10.1016/s0891-5849(02)01356-4 12614843

[B10] Brown D. A. Perry J. B. Allen M. E. Sabbah H. N. Stauffer B. L. Shaikh S. R. (2017). Expert consensus document: mitochondrial function as a therapeutic target in heart failure. *Nat. Rev. Cardiol.* 14 238–250. 10.1038/nrcardio.2016.203 28004807 PMC5350035

[B11] Brown K. Xie S. Qiu X. Mohrin M. Shin J. Liu Y. (2013). SIRT3 reverses aging-associated degeneration. *Cell Rep.* 3 319–327. 10.1016/j.celrep.2013.01.005 23375372 PMC3582834

[B12] Brown K. A. Didion S. P. Andresen J. J. Faraci F. M. (2007). Effect of aging, MnSOD deficiency, and genetic background on endothelial function: evidence for MnSOD haploinsufficiency. *Arterioscler. Thromb. Vasc. Biol.* 27 1941–1946. 10.1161/atvbaha.107.146852 17556650

[B13] Chaudhary K. R. El-Sikhry H. Seubert J. M. (2011). Mitochondria and the aging heart. *J. Geriatr. Cardiol.* 8 159–167. 10.3724/sp.j.1263.2011.00159 22783302 PMC3390067

[B14] Chen H. Wang X. Han J. Fan Z. Sadia S. Zhang R. (2017). AKT and its related molecular feature in aged mice skin. *PLoS One* 12:e0178969. 10.1371/journal.pone.0178969 28591208 PMC5462418

[B15] Chen T. Liu J. Li N. Wang S. Liu H. Li J. (2015). Mouse SIRT3 attenuates hypertrophy-related lipid accumulation in the heart through the deacetylation of LCAD. *PLoS One* 10:e0118909. 10.1371/journal.pone.0118909 25748450 PMC4351969

[B16] Chen Y.-R. Zweier J. L. (2014). Cardiac mitochondria and reactive oxygen species generation. *Circ. Res.* 114 524–537. 10.1161/CIRCRESAHA.114.300559 24481843 PMC4118662

[B17] Cheng S. Xanthakis V. Sullivan L. M. Lieb W. Massaro J. Aragam J. (2010). Correlates of echocardiographic indices of cardiac remodeling over the adult life course: longitudinal observations from the Framingham Heart Study. *Circulation* 122 570–578. 10.1161/CIRCULATIONAHA.110.937821 20660804 PMC2942081

[B18] Chiao Y. A. Rabinovitch P. S. (2015). The aging heart. *Cold Spring Harb. Perspect. Med.* 5:a025148. 10.1101/cshperspect.a025148 26328932 PMC4561390

[B19] Colom B. Oliver J. Roca P. Garcia-Palmer F. J. (2007). Caloric restriction and gender modulate cardiac muscle mitochondrial H2O2 production and oxidative damage. *Cardiovasc. Res.* 74 456–465. 10.1016/j.cardiores.2007.02.001 17376413

[B20] Dai D.-F. Rabinovitch P. S. (2009). Cardiac aging in mice and humans: the role of mitochondrial oxidative stress. *Trends Cardiovasc. Med.* 19 213–220. 10.1016/j.tcm.2009.12.004 20382344 PMC2858758

[B21] Darwesh A. M. Sosnowski D. K. Lee T. Y. T. Keshavarz-Bahaghighat H. Seubert J. M. (2019). Insights into the cardioprotective properties of n-3 PUFAs against ischemic heart disease via modulation of the innate immune system. *Chem. Biol. Interact.* 308 20–44. 10.1016/j.cbi.2019.04.037 31067438

[B22] Decker M. Adamska M. Cronin A. Giallonardo F. D. Burgener J. Marowsky A. (2012). EH3 (ABHD9): the first member of a new epoxide hydrolase family with high activity for fatty acid epoxides. *J. Lipid Res.* 53 2038–2045. 10.1194/jlr.M024448 22798687 PMC3435537

[B23] Edin M. L. Hamedani B. G. Gruzdev A. Graves J. P. Lih F. B. Arbes S. J. (2018). Epoxide hydrolase 1 (EPHX1) hydrolyzes epoxyeicosanoids and impairs cardiac recovery after ischemia. *J. Biol. Chem.* 293 3281–3292. 10.1074/jbc.RA117.000298 29298899 PMC5836130

[B24] Fava C. Montagnana M. Danese E. Almgren P. Hedblad B. Engstrom G. (2010). Homozygosity for the EPHX2 K55R polymorphism increases the long-term risk of ischemic stroke in men: a study in Swedes. *Pharmacogenet. Genomics* 20 94–103. 10.1097/FPC.0b013e3283349ec9 20065888

[B25] Fedorova M. Bollineni R. C. Hoffmann R. (2014). Protein carbonylation as a major hallmark of oxidative damage: update of analytical strategies. *Mass Spectrom. Rev.* 33 79–97. 10.1002/mas.21381 23832618

[B26] Grapo J. D. McCord J. M. Fridovich I. (1978). Preparation and assay of superoxide dismutase. *Meth. Enzymol.* 53 382–393. 362127 10.1016/s0076-6879(78)53044-9

[B27] Hafner A. V. Dai J. Gomes A. P. Xiao C.-Y. Palmeira C. M. Rosenzweig A. (2010). Regulation of the mPTP by SIRT3-mediated deacetylation of CypD at lysine 166 suppresses age-related cardiac hypertrophy. *Aging* 2 914–923. 10.18632/aging.100252 21212461 PMC3034180

[B28] Harris T. R. Hammock B. D. (2013). Soluble epoxide hydrolase: gene structure, expression and deletion. *Gene* 526 61–74. 10.1016/j.gene.2013.05.008 23701967 PMC3733540

[B29] He J. Wang C. Zhu Y. Ai D. (2016). Soluble epoxide hydrolase: a potential target for metabolic diseases: 可溶性表氧化物酶: 代谢性疾病的潜在治疗靶点. *J. Diabetes* 8 305–313. 10.1111/1753-0407.12358 26621325

[B30] Hebert A. S. Dittenhafer-Reed K. E. Yu W. Bailey D. J. Selen E. S. Boersma M. D. (2013). Calorie restriction and SIRT3 trigger global reprogramming of the mitochondrial protein acetylome. *Mol. Cell* 49 186–199. 10.1016/j.molcel.2012.10.024 23201123 PMC3704155

[B31] Hua Y. Zhang Y. Ceylan-Isik A. F. Wold L. E. Nunn J. M. Ren J. (2011). Chronic Akt activation accentuates aging-induced cardiac hypertrophy and myocardial contractile dysfunction: role of autophagy. *Basic Res. Cardiol.* 106 1173–1191. 10.1007/s00395-011-0222-8 21901288

[B32] Huang A. Kaley G. (2004). Gender-specific regulation of cardiovascular function: estrogen as key player. *Microcirculation* 11 9–38. 10.1080/10739680490266162 15280095

[B33] Imig J. D. (2006). Cardiovascular therapeutic aspects of soluble epoxide hydrolase inhibitors. *Cardiovasc. Drug Rev.* 24 169–188. 10.1111/j.1527-3466.2006.00169.x 16961727

[B34] Imig J. D. Hammock B. D. (2009). Soluble epoxide hydrolase as a therapeutic target for cardiovascular diseases. *Nat. Rev. Drug Discov.* 8 794–805. 10.1038/nrd2875 19794443 PMC3021468

[B35] Jamieson K. L. Endo T. Darwesh A. M. Samokhvalov V. Seubert J. M. (2017a). Cytochrome P450-derived eicosanoids and heart function. *Pharmacol. Ther.* 179 47–83. 10.1016/j.pharmthera.2017.05.005 28551025

[B36] Jamieson K. L. Samokhvalov V. Akhnokh M. K. Lee K. Cho W. J. Takawale A. (2017b). Genetic deletion of soluble epoxide hydrolase provides cardioprotective responses following myocardial infarction in aged mice. *Prostaglandins Other Lipid Mediat.* 132 47–58. 10.1016/j.prostaglandins.2017.01.001 28104457

[B37] Justo R. Frontera M. Pujol E. Rodríguez-Cuenca S. Lladó I. Garc F. J. (2005). Gender-related differences in morphology and thermogenic capacity of brown adipose tissue mitochondrial subpopulations. *Life Sci.* 76 1147–1158. 10.1016/j.lfs.2004.08.019 15620578

[B38] Keller K. M. Howlett S. E. (2016). Sex differences in the biology and pathology of the aging heart. *Can. J. Cardiol.* 32 1065–1073. 10.1016/j.cjca.2016.03.017 27395082

[B39] Kincaid B. Bossy-Wetzel E. (2013). Forever young: SIRT3 a shield against mitochondrial meltdown, aging, and neurodegeneration. *Front. Aging Neurosci.* 5:48. 10.3389/fnagi.2013.00048 24046746 PMC3764375

[B40] Koentges C. Bode C. Bugger H. (2016). SIRT3 in cardiac physiology and disease. *Front. Cardiovasc. Med.* 3:38. 10.3389/fcvm.2016.00038 27790619 PMC5061741

[B41] Kong X. Wang R. Xue Y. Liu X. Zhang H. Chen Y. (2010). Sirtuin 3, a new target of PGC-1alpha, plays an important role in the suppression of ROS and mitochondrial biogenesis. *PLoS One* 5:e11707. 10.1371/journal.pone.0011707 20661474 PMC2908542

[B42] Krumholz H. M. Larson M. Levy D. (1993). Sex differences in cardiac adaptation to isolated systolic hypertension. *Am. J. Cardiol.* 72 310–313. 10.1016/0002-9149(93)90678-6 8342510

[B43] Larsen S. Nielsen J. Hansen C. N. Nielsen L. B. Wibrand F. Stride N. (2012). Biomarkers of mitochondrial content in skeletal muscle of healthy young human subjects. *J. Physiol.* 590 3349–3360. 10.1113/jphysiol.2012.230185 22586215 PMC3459047

[B44] Lee J. Dahl M. Grande P. Tybjærg-Hansen A. Nordestgaard B. G. (2010). Genetically reduced soluble epoxide hydrolase activity and risk of stroke and other cardiovascular disease. *Stroke* 41 27–33. 10.1161/STROKEAHA.109.567768 19940276

[B45] Li Y. Huang T.-T. Carlson E. J. Melov S. Ursell P. C. Olson J. L. (1995). Dilated cardiomyopathy and neonatal lethality in mutant mice lacking manganese superoxide dismutase. *Nat. Genet.* 11 376–381. 10.1038/ng1295-376 7493016

[B46] Marowsky A. Burgener J. Falck J. R. Fritschy J. M. Arand M. (2009). Distribution of soluble and microsomal epoxide hydrolase in the mouse brain and its contribution to cerebral epoxyeicosatrienoic acid metabolism. *Neuroscience* 163 646–661. 10.1016/j.neuroscience.2009.06.033 19540314

[B47] Marowsky A. Haenel K. Bockamp E. Heck R. Rutishauser S. Mule N. (2016). Genetic enhancement of microsomal epoxide hydrolase improves metabolic detoxification but impairs cerebral blood flow regulation. *Arch. Toxicol.* 90 3017–3027. 10.1007/s00204-016-1666-2 26838043 PMC5104800

[B48] Martín-Fernández B. Gredilla R. (2016). Mitochondria and oxidative stress in heart aging. *Age* 38 225–238. 10.1007/s11357-016-9933-y 27449187 PMC5061683

[B49] Matsushima S. Sadoshima J. (2015). The role of sirtuins in cardiac disease. *Am. J. Physiol. Heart Circ. Physiol.* 309 H1375–H1389. 10.1152/ajpheart.00053.2015 26232232 PMC4666968

[B50] Merz A. A. Cheng S. (2016). Sex differences in cardiovascular ageing. *Heart* 102 825–831. 10.1136/heartjnl-2015-308769 26917537 PMC5993677

[B51] Monti J. Fischer J. Paskas S. Heinig M. Schulz H. Gösele C. (2008). Soluble epoxide hydrolase is a susceptibility factor for heart failure in a rat model of human disease. *Nat. Genet.* 40 529–537. 10.1038/ng.129 18443590 PMC7370537

[B52] Nithipatikom K. Endsley M. P. Pfeiffer A. W. Falck J. R. Campbell W. B. (2014). A novel activity of microsomal epoxide hydrolase: metabolism of the endocannabinoid 2-arachidonoylglycerol. *J. Lipid Res.* 55 2093–2102. 10.1194/jlr.M051284 24958911 PMC4174002

[B53] North B. J. Sinclair D. A. (2012). The intersection between aging and cardiovascular disease. *Circ. Res.* 110 1097–1108. 10.1161/CIRCRESAHA.111.246876 22499900 PMC3366686

[B54] Paneni F. Cañestro C. D. Libby P. Lüscher T. F. Camici G. G. (2017). The aging cardiovascular system: understanding it at the cellular and clinical levels. *J. Am. Coll. Cardiol.* 69 1952–1967. 28408026 10.1016/j.jacc.2017.01.064

[B55] Panth N. Paudel K. R. Parajuli K. (2016). Reactive oxygen species: a key hallmark of cardiovascular disease. *Adv. Med.* 2016:9152732. 27774507 10.1155/2016/9152732 PMC5059509

[B56] Parker B. A. Kalasky M. J. Proctor D. N. (2010). Evidence for sex differences in cardiovascular aging and adaptive responses to physical activity. *Eur. J. Appl. Physiol.* 110 235–246. 10.1007/s00421-010-1506-7 20480371 PMC2929283

[B57] Parodi-Rullán R. M. Chapa-Dubocq X. R. Javadov S. (2018). Acetylation of mitochondrial proteins in the heart: the role of SIRT3. *Front. Physiol.* 9:1094. 10.3389/fphys.2018.01094 30131726 PMC6090200

[B58] Pillai V. B. Samant S. Sundaresan N. R. Raghuraman H. Kim G. Bonner M. Y. (2015). Honokiol blocks and reverses cardiac hypertrophy in mice by activating mitochondrial Sirt3. *Nat. Commun.* 6:6656. 10.1038/ncomms7656 25871545 PMC4441304

[B59] Pillai V. B. Sundaresan N. R. Gupta M. P. (2014). Regulation of Akt signaling by sirtuins: its implication in cardiac hypertrophy and aging. *Circ. Res.* 114 368–378. 10.1161/CIRCRESAHA.113.300536 24436432 PMC4228987

[B60] Pinot F. Grant D. F. Spearow J. L. Parker A. G. Hammock B. D. (1995). Differential regulation of soluble epoxide hydrolase by clofibrate and sexual hormones in the liver and kidneys of mice. *Biochem. Pharmacol.* 50 501–508. 10.1016/0006-2952(95)00167-x 7646556

[B61] Poljsak B. Milisav I. (2013). “Aging, oxidative stress and antioxidants,” in *Oxidative Stress and Chronic Degenerative Diseases-A Role for Antioxidants*, ed. Morales-Gonzalez J. A., (London: Intechopen), 331–353.

[B62] Porter G. A. Urciuoli W. R. Brookes P. S. Nadtochiy S. M. (2014). SIRT3 deficiency exacerbates ischemia-reperfusion injury: implication for aged hearts. *Am. J. Physiol. Heart Circ. Physiol.* 306 H1602–H1609. 10.1152/ajpheart.00027.2014 24748594 PMC4059981

[B63] Qin J. Le Y. Froogh G. Kandhi S. Jiang H. Luo M. (2016). Sexually dimorphic adaptation of cardiac function: roles of epoxyeicosatrienoic acid and peroxisome proliferator-activated receptors. *Physiol. Rep.* 4:e12838. 10.14814/phy2.12838 27354541 PMC4923237

[B64] Regitz-Zagrosek V. Kararigas G. (2016). Mechanistic pathways of sex differences in cardiovascular disease. *Physiol. Rev.* 97 1–37. 10.1152/physrev.00021.2015 27807199

[B65] Seubert J. M. Sinal C. J. Graves J. DeGraff L. M. Bradbury J. A. Lee C. R. (2006). Role of soluble epoxide hydrolase in postischemic recovery of heart contractile function. *Circ. Res.* 99 442–450. 10.1161/01.res.0000237390.92932.37 16857962 PMC2072806

[B66] Shaw L. J. Bugiardini R. Merz C. N. B. (2009). Women and ischemic heart disease: evolving knowledge. *J. Am. Coll. Cardiol.* 54 1561–1575. 10.1016/j.jacc.2009.04.098 19833255 PMC2789479

[B67] Shuey M. M. Billings F. T. T. Wei S. Milne G. L. Nian H. Yu C. (2017). Association of gain-of-function EPHX2 polymorphism Lys55Arg with acute kidney injury following cardiac surgery. *PLoS One* 12:e0175292. 10.1371/journal.pone.0175292 28552948 PMC5446112

[B68] Siasos G. Tsigkou V. Kosmopoulos M. Theodosiadis D. Simantiris S. Tagkou N. M. (2018). Mitochondria and cardiovascular diseases—from pathophysiology to treatment. *Ann. Transl. Med.* 6:256. 30069458 10.21037/atm.2018.06.21 PMC6046286

[B69] Sinal C. J. Miyata M. Tohkin M. Nagata K. Bend J. R. Gonzalez F. J. (2000). Targeted disruption of soluble epoxide hydrolase reveals a role in blood pressure regulation. *J. Biol. Chem.* 275 40504–40510. 10.1074/jbc.m008106200 11001943

[B70] Singh S. P. Schragenheim J. Cao J. Falck J. R. Abraham N. G. Bellner L. (2016). PGC-1 alpha regulates HO-1 expression, mitochondrial dynamics and biogenesis: role of epoxyeicosatrienoic acid. *Prostaglandins Other Lipid Mediat.* 125 8–18. 10.1016/j.prostaglandins.2016.07.004 27418542 PMC5536246

[B71] Spector A. A. Norris A. W. (2007). Action of epoxyeicosatrienoic acids on cellular function. *Am. J. Physiol. Cell Physiol.* 292 C996–C1012. 16987999 10.1152/ajpcell.00402.2006

[B72] Spinazzi M. Casarin A. Pertegato V. Salviati L. Angelini C. (2012). Assessment of mitochondrial respiratory chain enzymatic activities on tissues and cultured cells. *Nat. Protoc.* 7 1235–1246. 10.1038/nprot.2012.058 22653162

[B73] Steenman M. Lande G. (2017). Cardiac aging and heart disease in humans. *Biophys. Rev.* 9 131–137. 10.1007/s12551-017-0255-9 28510085 PMC5418492

[B74] Sundaresan N. R. Bindu S. Pillai V. B. Samant S. Pan Y. Huang J.-Y. (2016). SIRT3 blocks aging-associated tissue fibrosis in mice by deacetylating and activating glycogen synthase kinase 3β. *Mol. Cell. Biol.* 36 678–692. 10.1128/mcb.00586-15 26667039 PMC4760222

[B75] Sundaresan N. R. Gupta M. Kim G. Rajamohan S. B. Isbatan A. Gupta M. P. (2009). Sirt3 blocks the cardiac hypertrophic response by augmenting Foxo3a-dependent antioxidant defense mechanisms in mice. *J. Clin. Invest.* 119 2758–2771. 10.1172/JCI39162 19652361 PMC2735933

[B76] Wei T. Huang G. Gao J. Huang C. Sun M. Wu J. (2017). Sirtuin 3 deficiency accelerates hypertensive cardiac remodeling by impairing angiogenesis. *J. Am. Heart Assoc.* 6:e006114. 10.1161/JAHA.117.006114 28862956 PMC5586452

[B77] Wenger N. K. (2012). Women and coronary heart disease: a century after Herrick: understudied, underdiagnosed, and undertreated. *Circulation* 126 604–611. 10.1161/circulationaha.111.086892 22850362

[B78] Whitehead J. C. Hildebrand B. A. Sun M. Rockwood M. R. Rose R. A. Rockwood K. (2014). A clinical frailty index in aging mice: comparisons with frailty index data in humans. *J. Gerontol. A Biol. Sci. Med. Sci.* 69 621–632. 10.1093/gerona/glt136 24051346 PMC4022099

[B79] Yang Y.-M. Sun D. Kandhi S. Froogh G. Zhuge J. Huang W. (2018). Estrogen-dependent epigenetic regulation of soluble epoxide hydrolase via DNA methylation. *Proc. Natl. Acad. Sci. U.S.A.* 115 613–618. 10.1073/pnas.1716016115 29295935 PMC5776989

[B80] Zeng L. Yang Y. Hu Y. Sun Y. Du Z. Xie Z. (2014). Age-related decrease in the mitochondrial sirtuin deacetylase Sirt3 expression associated with ROS accumulation in the auditory cortex of the mimetic aging rat model. *PLoS One* 9:e88019. 10.1371/journal.pone.0088019 24505357 PMC3913718

[B81] Zhang W. Davis C. M. Edin M. L. Lee C. R. Zeldin D. C. Alkayed N. J. (2013). Role of endothelial soluble epoxide hydrolase in cerebrovascular function and ischemic injury. *PLoS One* 8:e61244. 10.1371/journal.pone.0061244 23585883 PMC3621731

[B82] Zhang W. Otsuka T. Sugo N. Ardeshiri A. Alhadid Y. K. Iliff J. J. (2008). Soluble epoxide hydrolase gene deletion is protective against experimental cerebral ischemia. *Stroke* 39 2073–2078. 10.1161/STROKEAHA.107.508325 18369166 PMC2654189

[B83] Zhao B. Sun L. K. Jiang X. Zhang Y. Kang J. Meng H. (2019). Genipin protects against cerebral ischemia-reperfusion injury by regulating the UCP2-SIRT3 signaling pathway. *Eur. J. Pharmacol.* 845 56–64. 10.1016/j.ejphar.2018.12.028 30582911

[B84] Zhou T.-J. Gao Y. (2010). Molecular mechanisms of cardiac aging. *J. Geriatr. Cardiol.* 7 184–188.

[B85] Zhu X. L. Wang L. Wang Z. Chen S. Z. Zhang W. Q. Ma M. M. (2015). Relationship between EPHX2 gene polymorphisms and essential hypertension in Uygur, Kazakh, and Han. *Genet. Mol. Res.* 14 3474–3480. 10.4238/2015.April.15.11 25966114

